# The Trypanosomal Transferrin Receptor of Trypanosoma Brucei—A Review

**DOI:** 10.3390/tropicalmed4040126

**Published:** 2019-10-01

**Authors:** Christopher K. Kariuki, Benoit Stijlemans, Stefan Magez

**Affiliations:** 1 Laboratory of Cellular and Molecular Interactions (CMIM), Vrije Universiteit Brussels, Brussels, 1050 Ixelles, Belgium; Benoit.Stijlemans@vub.be; 2 Department of Tropical and Infectious Diseases, Institute of Primate Research (IPR), 00502 Nairobi, Kenya; 3 Myeloid Cell Immunology Lab, VIB Center for Inflammation Research, Brussels, 9052 Gent, Belgium; 4 Laboratory for Biomedical Research, Ghent University Global Campus, Yeonsu-Gu, Incheon 219220, Korea

**Keywords:** trypanosomosis, iron, transferrin, transferrin receptor, nutritional immunity, flagellar pocket

## Abstract

Iron is an essential element for life. Its uptake and utility requires a careful balancing with its toxic capacity, with mammals evolving a safe and bio-viable means of its transport and storage. This transport and storage is also utilized as part of the iron-sequestration arsenal employed by the mammalian hosts’ ‘nutritional immunity’ against parasites. Interestingly, a key element of iron transport, i.e., serum transferrin (Tf), is an essential growth factor for parasitic haemo-protozoans of the genus *Trypanosoma*. These are major mammalian parasites causing the diseases human African trypanosomosis (HAT) and animal trypanosomosis (AT). Using components of their well-characterized immune evasion system, bloodstream *Trypanosoma brucei* parasites adapt and scavenge for the mammalian host serum transferrin within their broad host range. The expression site associated genes (ESAG6 and 7) are utilized to construct a heterodimeric serum Tf binding complex which, within its niche in the flagellar pocket, and coupled to the trypanosomes’ fast endocytic rate, allows receptor-mediated acquisition of essential iron from their environment. This review summarizes current knowledge of the trypanosomal transferrin receptor (TfR), with emphasis on the structure and function of the receptor, both in physiological conditions as well as in conditions where the iron supply to parasites is being limited. Potential applications using current knowledge of the parasite receptor are also briefly discussed, primarily focused on potential therapeutic interventions.

## 1. Trypanosomes and Their Need for Iron during a Mammalian Infection

Iron is an essential requirement for life [1,2,3]. Iron’s biological utility lies in its cycling between two oxidation states, namely ferrous (Fe^2+^) and ferric (Fe^3+^) [4]. Thus, iron can serve as redox catalyst, which accepts and donates electrons [4]. As a result of this redox capability, once absorbed from the environment via the mammalian duodenum, iron is not circulated freely in mammalian tissues, as it readily catalyzes conversion of H_2_O_2_ into toxic free radicals via the Fenton reaction [1,5].

Under conditions of neutral pH and high oxygen tension of most physiological fluids, such as mammalian serum, iron exists predominantly in its ferric (Fe^3+^) form [3]. Given its high hydrolytic propensity, under these conditions, ferric iron (Fe^3+^) in excess of 2.5 × 10^−18^ M readily polymerizes, resulting in an insoluble and bio-inert form [6]. Therefore, to keep ferric iron (Fe^3+^) soluble, bio-available, and render it non-toxic, vertebrates such as mammals store and transport iron via specific iron sequestering molecules [3,6,7]. Storage of iron is achieved using two formats; in an soluble form as a mobilizable reserve by ferritin and an insolubly form as hemosiderin [3,8]. Transport of ferric iron (Fe^3+^) in the mammalian serum is usually as a tight, but reversible association, with an abundant serum carrier protein family, the transferrins [3,7,9,10].

Serum transferrin/serotransferrin (Tf) is the transporter of ferric iron (Fe^3+^) in the blood serum of vertebrates, acting as the connection between the ferritin storing hepatocytes to the diverse cellular population of the vertebrate body [9,10]. Serum transferrin, i.e., a 80 kDa glycoprotein, has been structurally resolved, indicating a bi-lobed tertiary structure (N- and C-lobes) with a short connecting loop between them as well as possessing two domains per lobe, with the ferric iron (Fe^3+^) binding sites located within the inter-domain clefts of each lobe [11]. In the presence of an anion, e.g., bicarbonate or carbonate, and at a physiological serum pH range, serum transferrin can bind either mono- or di-ferric iron atoms, transforming from apo- (iron-free) to holo- (iron-laden) transferrin [3,11]. Though both lobes bind ferric iron (Fe^3+^), there is a difference in binding capability as the C-terminal lobe binds Fe^3+^ more tightly and releases it more slowly [12]. Mammalian serum Tf is expressed in the liver, central nervous system (CNS), reproductive organs, spleen, and kidneys [13].

The rich resource of bio-available iron is a prized target for parasites, particularly those that reside in the bloodstream, such as the trypanosome species, *T. brucei*, thus leading to the diseases, human African trypanosomosis (HAT) and animal trypanosomosis (AT).

Animal trypanosomosis (also known as animal trypanosomiasis) is a parasitic disease of veterinary importance in the tropical world [14]. From its cradle in Africa, through the steppes of Asia to the far ends of the Americas, the disease has devastatingly negative economic and societal impacts [15,16]. 

**Figure 1:**
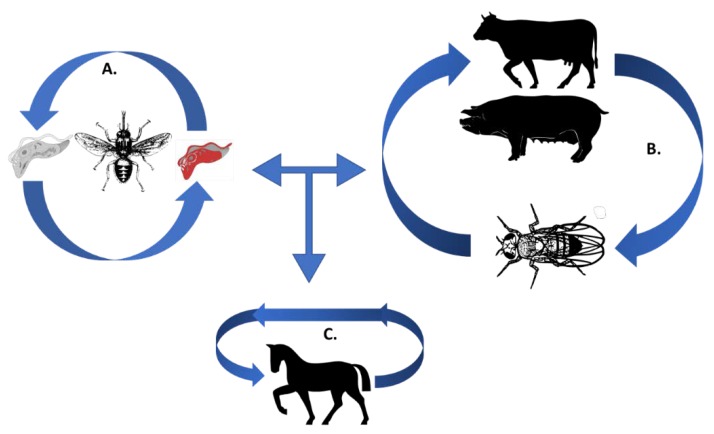
The trypanosomes’ lifecycle. (**A**) Cyclical transmission of *T. brucei* and *T. congolense* (causing ‘Nagana’) occurs in the Tsetse fly (*Glossina* species), in grey is a representation of the procyclic form and in red is the representation of the bloodstream form (BSF); (**B**) mechanical transmission via tabanids leading to trypanosomosis caused by *T. vivax* and *T. evansi*; (**C**) sexual transmission occurs in equines for *T. equiperdum* during the course of dourine [26,27,28].

In domestic livestock, AT is a wasting disease assigned by various names such as ‘*Nagana*’, ‘*Derrengadera*’, ‘*Dourine*’, ‘*Mals de coits*’, or ‘*Surra*’, depending on the causative *Trypanosoma* species [17]. Five of the most important species, namely, *T. vivax*, *T. evansi*, *T. congolense*, *T. equiperdum*, and *T. brucei* cause AT in all domestic animals [16]. These trypanosome species are considered heteroxenous (Figure 1) [18]. Of note, the *T. brucei* (sub-genus *Trypanozoon*) clade can be further divided into strictly animal infecting parasites (*T. brucei brucei*) and parasites able to infect also humans and higher primates, namely the zoonotic *T. brucei rhodesiense* and the anthroponotic *T. brucei gambiense* [19,20]. This is attributed to the fact that these latter parasites acquired the ability to resist trypanolytic molecular complexes, expressed by humans and higher primates as part of the innate immune system [20,21,22,23,24]. Due to this reason, and its implications for human medicine, the *T. brucei* clade has received greater attention and is better characterized, despite being it having a lower, though still potent, worldwide impact on veterinary economy, than for example *T. congolense* (Sub-genus *Nannomonas*) or *T vivax* (Sub-genus *Dutonella*) [25].

By parasitizing the haemo-lymphatic environment, trypanosomes must reconcile two seemingly conflicting requirements, namely, to avoid the immune responses of the mammalian host by rapid variation of their plasma membrane as well as efficiently acquire potentially scarce nutritive resources from their environment via the same plasma membrane [29].

## 2. Expression Sites; the Trypanosomal Swiss Army Knife for Host Adaptation

Trypanosomes exemplify the general survival strategy of phenotypic variation, a mechanism by which diverse parasitic organisms, from viruses to eukaryotes, contain a subset of contingency genes hypermutating as a rapid adaptation to hostile or changing environments [30]. This is the classically described paradigm of antigenic variation of the major trypanosome surface glycoproteins, the variable surface glycoprotein (VSG) [31,32]. Antigenic variation in trypanosomes occurs when a subset of a trypanosome population randomly switches the expression of a particular set of VSG genes, thus displaying a different surface coat of VSGs [29,30,33]. Contrary to avoiding the mammalian hosts’ immune system, VSGs are highly immunogenic, with surface epitopes that are highly recognizable by the mammalian hosts’ immune system [29]. When the immune system clears the parasite population bearing the recognized VSG by the humoral response, there is the emergence of sub-population of the haemo-protozoans with a different surface coat of VSG homodimers, against which the immune system has to prepare another humoral response [30]. The ebb and rise of different populations of trypanosomes is reflected on the parasitemia pattern observed microscopically and confers to the parasite an advantage of establishing a controlled but chronic host infection [29]. 

**Figure 2:**
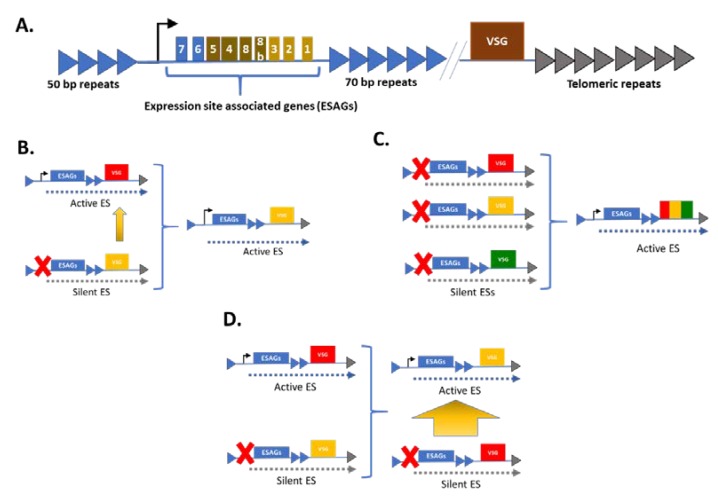
Modes of variable surface glycoprotein (VSG) gene switching. (**A**) The expression site (ES) of VSG including the proximal promoter, expression site-associated genes (ESAGs), the VSG, and their associated repeats. Transcription occurs in a polycistronic manner, with the ESAGs 7 and 6 being most proximally located and the VSG most distally located on the expression sites. (**B**) Mechanism of gene conversion: A VSG gene conversion event occurs when a VSG gene from a silent ES is copied (via homologous recombination) into an active ES where it is expressed. (**C**) Mechanism of segmental gene conversion: Various VSG gene recombination events occurring in the silent ES, leading to formation of a novel and mosaic VSG gene, which is copied via homologous recombination into the active ES. (**D**) Mechanism of transcriptional switching (in-situ (in)-activation): A non-recombination event occurs that activates a new (previously silent) ES while inactivating a previously active ES [48].

The active *T. brucei* spp. bloodstream form (BSF) VSG gene is obtained from an arsenal of more than 1500 VSG genes, most of which are pseudogenes in sub-telomeric silent arrays [34,35,36]. For expression, the VSG gene has to be relocated to a devoted genomic environment, aptly termed the expression site (ES), at the telomeric regions of one of the large chromosomes [37,38,39,40]. In the ES, specifically the bloodstream form expression site (BES), transcription occurs in a polycistronic manner, with VSG genes always the last gene of the unit, separated from the other genes, by 70 bp repeats (Figure 2A) [39,41,42]. The polycistronic nature of transcription allows a tightly regulated expression of the VSG gene from only 1 of the 20 telomeric expression sites (ESs), in conjunction with associated proteins within the ES, namely the expression site associated genes (ESAGs) [35,43]. The polycistronic mRNA is transcribed from a highly conserved BES promoter, that has also been shown to be sensitive to temperature changes, and which is considered a specificity signal that triggers the activation of the BES upon encountering the bloodstream of a mammalian host [42].

With only one type of VSG being expressed at a time, a portion of the trypanosome population is guaranteed invisibility from the host’s immune system at any given time [35,43,44,45,46,47]. Variation within the VSG is achieved by homologous DNA recombination such as gene conversion, targeted to the active VSG ESs (Figure 2B,C) or by either transcriptional switching (in-situ activation and in-activation (Figure 2D) between the VSG ESs) [40].

**Figure 3:**
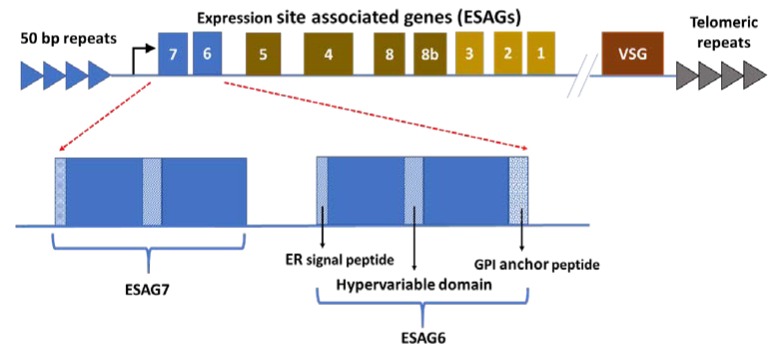
The ESAG 6 and 7 genes (forming the pESAG 6 and 7 heterodimeric transferrin receptor) are transcribed as part of the polycistronic VSG mRNA. The polycistronic VSG mRNA is transcribed from the active subtelomeric expression site (ES) (only 1 of the 20 available ES is active at a time). The gene products appear to have a similar structure except for the glycosyl phosphatidyl inositol (GPI) peptide on ESAG 6 [35,45,46,47].

This immune evasion tactic also serves a rather utilitarian purpose for the parasite. As stated previously, in addition to successfully evading the host’s immune system, the extracellular parasite must somehow combine the antigenic variation of its homogenous and over-arching surface coat, with the uptake of different substrates that it requires for its survival [49]. The parasite approaches this possible problem using a specific set of genes, namely the expression site associated genes (ESAGs). Within the *T. brucei* ES’s, the BSF trypanosome also has a more limited repertoire of ESAGs (Figure 3) [43]. Most of the ESAGs encode predicted proteins that contain N-terminal signal sequences as well as putative hydrophobic membrane spanning segments, indicative of surface exposed proteins, such as the integral membrane proteins such as the receptor-like transmembrane adenylyl cyclase (ESAG4), a surface transporter (ESAG10), and the glycosyl phosphatidyl inositol (GPI)-anchored heterodimeric trypanosomal transferrin receptor (ESAG6/7) and serum-associated resistance antigen (SRA) [50,51,52]. Several ESAGs have been found to belong to multigene families including pseudogenes and members that are not transcribed within the ESs, aptly named “genes related to ESAGs” (GRESAGs) [51]. In total, within the *T. brucei* clade, sequencing has revealed about 12 polymorphic genes comprising the ESAGs, and approximately 20 different variants of each ESAG [43,45,46,51,53]. In comparison, there are relatively fewer homologs or orthologs in the closely related *T. congolense* or *T. vivax* [54]. An analysis of the cell-surface phylome for the trypanosomes revealed that for some ESAGs, e.g., ESAG1, there are neither homologs nor orthologs, indicating recent innovation by the *T. brucei* clade [54]. Other ESAGs, for example the ESAG6 and 7, encoding the trypanosomal transferrin receptor and that are considered essential for bloodstream iron scavenging in the *T. brucei* spp., are missing from *T. vivax* but are present in *T. congolense*, which is indicative of a more recently shared ancestry between *T. congolense* and *T. brucei* [54]. 

Some genes in the expression site, i.e., ESAG 6, 7, and SRA, share an evolutionary origin with the VSGs, and may thus confer an increased capacity for the parasite to adapt to various mammalian hosts [51,52,55]. Given that some of the ESAGs are involved in substrate capture, it therefore seems plausible that the transcriptional switching between multiple expression sites would offer the parasite antigenic variation for these minor surface proteins [40,50].

More importantly, the transcriptional switching of BESs would also allow the selection and expression of the appropriate ESAG 6 and 7 genes for efficient capture of the requisite host transferrin molecule, which would enable the trypanosome to adapt not only to the different mammalian host range that is available to the *T. brucei* spp., but as well to the mammalian hosts’ “nutritional immunity” [56,57].

## 3. The Trypanosomal Transferrin Receptor: A Structural Review

As indicated previously, ESAG 6 and 7 genes (encoding the trypanosomal heterodimeric transferrin receptor) are transcribed in a polycistronic mRNA together with the current VSG from an upstream promoter (Figure 3) [58,59]. 

However, given that both ESAG 6 and 7 genes are situated nearest to the ES promoter site with the end of ESAG 6 being approximately +5.3 kB from this promoter, there is a low but detectable transcription occurring from ‘inactive’ ESs in bloodstream form (BSF) trypanosomes [60,61]. It has been estimated that up to 20% of ESAG 6 mRNA originates from ‘inactive’ ESs [60]. This promoter-proximal position of the two genes provides the parasite with a flexibility in the regulation of the genes, providing a competitive edge, especially during periods of limited transferrin uptake, e.g., during the switch to another host.

**Figure 4:**
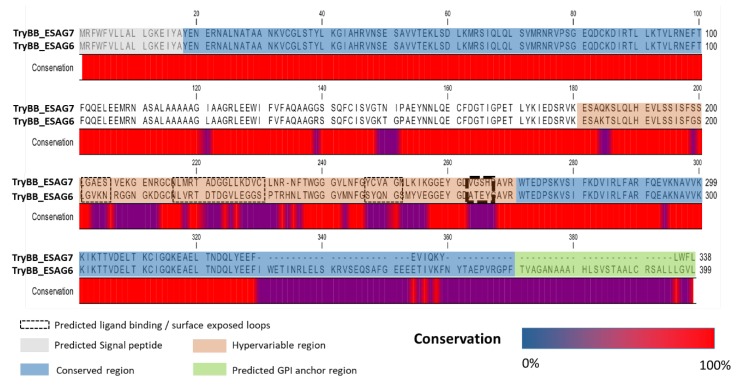
The products of ESAG 6 gene (pESAG6) and ESAG 7 (pESAG7) in *T. b. brucei* Strain 427 (UniProt ID Q8WPU1_9TRYP and Q8WPU2_9TRYP respectively) are similar along their N-terminus, differing only at their C-terminal end (GPI anchor peptide).

Different but homologous pESAG6-7 heterodimers encoded by the different ESs are present in the BSF trypanosomes (Figure 4), differing in sequence identity by only 1–10% [60]. The proteins pESAG6 and 7 have been shown to be synthesized individually, containing N-terminal signal sequences that are not present in the matured protein forms [62].

In *T. brucei*, the two genes (ESAG 6 and 7) give rise to heterogeneously glycosylated proteins between 50–60 kDa and 40–42 kDa, respectively [63,64]. Only heterodimeric complexes (with a 1:1 stoichiometry) of the products from the two genes form a functional *T. brucei* trypanosomal TfR, indicating that there is a combination of elements, specific to each subunit, that are required for the transferrin binding site [65,66]. Though the protein subunits are glycosylated, the pESAG 6/pESAG 7 heterodimer can function just as well without this post-translational modification [64]. Small amino acid switches in the surface exposed loops of the pESAG 6-pESAG 7 complex, forming the transferrin binding site, brings about differences in affinity of the various TfRs for their respective Tf ligands in different hosts (ranging from 2 nM to 1 µM), all lying within the reported physiological range of Tf (30–40 µM) in the mammalian serum [67]. This allows rapid adaptation of the parasites’ Tf scavenging capacity in different hosts, particularly in the presence of host antibodies [68,69,70]. This high affinity for Tf combined with the rapid recycling of the TbTfR enables the bloodstream parasites to actively compete, despite efforts by the mammalian host immune system, for the limited substrate until a novel higher affinity TfR is expressed [61].

The ESAG 6 and 7 products (pESAG6 and pESAG7, respectively) are nearly identical in sequence, especially in their N-termini, which also contain the ligand binding sites, differing only in their C-termini [65]. The two proteins also share significant homology (20% identity and 60% similarity) with an A domain type VSG N-terminus, indicating a possible structural design requirement for accommodation within the dense VSG protein coat as well as an evolutionary relationship between the two ESAGs and the VSG N-terminal domain [51,65,66,71]. The pESAG6 and 7 appear to have a number of the structurally conserved features of the N-terminus of the VSG class A, whereby, especially, the ESAG 7 gene appears to be a VSG gene conversion domain [66]. Salmon et al. [63] showed that the ESAG 7/6 can be aligned to VSG (sharing significant similarity) and hypothesized that the binding sites are most likely to occur on the surface exposed loops of the heterodimeric protein (Figure 4), i.e., in the dashed boxes (where they align with the surface exposed regions of VSG gene products). Sequence secondary structure predictions e.g., JPRED indicates that these regions are most likely in loops. Prediction of ligand binding regions using the Kolaskar and Tongaonkar method [64] indicates their surface accessibility (again confirming the VSG linkage). In fact, a VSG-based chimeric TfR has been constructed and shown to effectively bind Tf [66]. This was achieved by grafting the C-termini of either ESAG 6 or 7 with the N-terminus of a MiTat 1.5 VSG [66]. The heterogeneously expressed chimeric VSG-ESAG 6 and VSG-ESAG 7 gave a heterodimeric receptor that bound Tf equally well as the native pESAG6/7 heterodimer (1.2 ± 0.27 µM vs. 0.97 ± 0.36 µM, respectively) in *Xenopus* oocytes [65,66].

**Figure 5:**
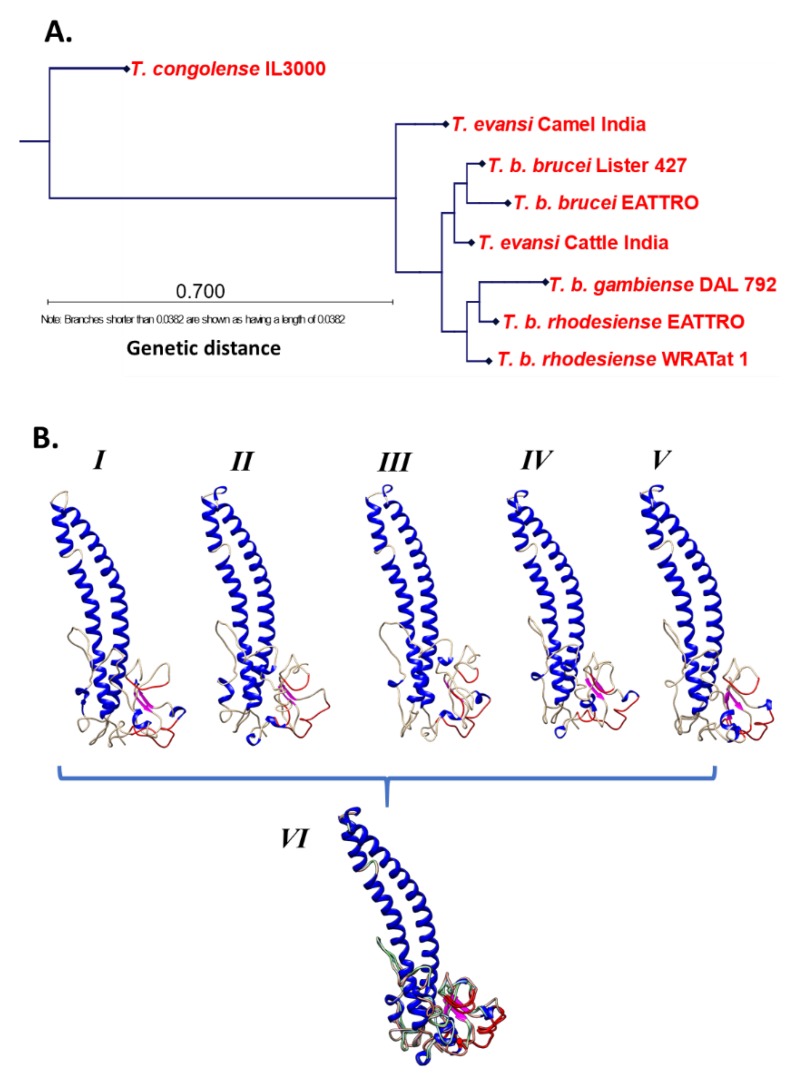
(**A**) The ESAG 6 gene is homologous within the *T. brucei* subspecies. A protein BLAST query for ESAG6 (which is the GPI-anchored partner of the ESAG7/6 heterodimer) revealed approximately 100 sequences from *T. brucei*, *T. evansi*, and *T. congolense*. The results were then assembled to make an alignment and a phylogenetic tree. Alignment was done using the program MUSCLE, and the phylogenetic tree was constructed via Neighbor joining algorithm with a JTT (Jones-Taylor-Thornton) protein substitution model: (**B**) Modelled ESAG6 is similarly structured between the different *T. brucei* subspecies i.e., I. *T. b. rhodesiense*, II. *T. b. gambiense*, III. *T. b. brucei*, IV. *T. evansi*, and V. *T. equiperdum*. (VI.) An overlay model built from a structural alignment of all the models indicates that the predicted binding site is on surface exposed loops (red ribbons) and occurs similarly in all the proteins. Helices are denoted by the blue ribbons, while the brown ribbons denote loops and the magenta ribbons denote beta sheets. Modelling was done on the *T. b. brucei* VSG ILTat 1.24 (PDB ID: 2VSG) using the SWISS-MODEL homology modelling server (https://swissmodel.expasy.org/) [75,76,77,78,79] with the 2.7 Å X-ray diffraction structure of ILTat 1.24 (2VSG.pdb) as a template [80].

The pESAG6 has a hydrophobic C-terminus that is eventually replaced by a GPI-anchor, making it the plasma-membrane bound member for the heterodimeric TfR [63,65,66,72]. An alignment of publicly available ESAG 6 gene sequences reveals the gene’s homology within the *T. brucei* subspecies (Figure 5A), particularly indicative of the reported close phylogenetic relation between *T. brucei* and *T. evansi* [25,73]. In contrast, the pESAG7 has no such modification, therefore it is hypothesized to bind non-covalently with the ESAG6 [63]. Between the two proteins, there are differences in residues within the stretches forming the ligand binding site, i.e., positions 205–215 and 223–238 of pESAG6 and 7, respectively [65,66]. These amino acid stretches, especially on pESAG6, have been mapped to surface exposed loops by modeling on the resolved N-terminal VSG crystal structure (Figure 5B) [66]. Switching, by site-directed mutagenesis, of key amino acid within these stretches of pESAG6 and 7, resulted in a predicted enhancement of the Tf binding capacity [66]. Further proof was obtained from the site-directed mutagenesis of residues immediately outside each of the four domains, which resulted in loss of the Tf binding capacity, predictably due to loss of the surface exposed loop structure [66]. Despite the endeavors to model the TfR by various groups [66,74], there has been no actual structure (via X-ray crystallography or NMR) resolved yet, not for the pESAG6 neither the pESAG 7 nor the pESAG6/7 heterodimer.

## 4. Fishing from a Hole; the Flagellar Pocket and the Quest for Iron

Iron is already a tightly controlled resource within the mammalian body fluids, with iron chelation molecules, i.e., serum transferrin (in blood and lymph) and lactoferrin (in external secretions), restricting the amount of bio-available ferric iron (Fe^3+^) in body fluids to about 10^−18^ M [81,82]. 

Iron availability is a key component employed by mammals to minimize the parasite burden and increase the hosts’ fitness [83]. By coupling of the mammalian immunosurveillance apparatus to iron metabolism, immunocompetence is associated with iron regulation [56,83]. Thus, the presence of parasites, indicated by their concomitant biochemical signals, signals a hazard to the mammalian system leading to the triggering of the acute-phase immune response [56]. A consequence of this is the sequestration of iron, thus limiting the bio-availability of this essential nutrient for circulating pathogens, a host-defense strategy known as ‘nutritional immunity’ [56]. Additionally, this also serves in strengthening specific immune effector mechanisms including the proliferation and functionality of immune cells, activation of cytokines, nitric oxide (NO) formation, activation of cellular proteins/peptides, and hormones that are dependent on iron availability [84,85]. 

Therefore, to sidestep nutritional immunity and obtain ferric iron (Fe^3+^) from their hosts body fluids, parasites have to either compete against these chelates by devising their own iron chelation molecules, e.g., bacterial siderophores, or cleave the mammalian iron chelates by releasing proteases, e.g., bacterial reductases, or scavenge for these chelates by using specific receptors, e.g., trypanosomal transferrin receptors [63].

Transferrin (Tf) acquisition is the main route of iron uptake for BSF trypanosomes, particularly *T. brucei* spp., which are exclusively extracellular within the bloodstream and which has been the model organism for studying Tf uptake [13,57,65,86,87,88]. Tf uptake has been shown to be saturable, indicative of receptor mediated endocytosis (RME) with the ligand in this case, holo-/apo-Tf, being specifically competed out from its receptor, the trypanosomal TfR [57,65].

Binding of Tf and recycling of the trypanosomal TfR occurs in a process quite different to that observed in mammalian cells (Table 1) [7,89]. The main route of iron uptake in trypanosomes is localized within the trypanosomal flagellar pocket (FP) [57,90]. 

**Figure 6:**
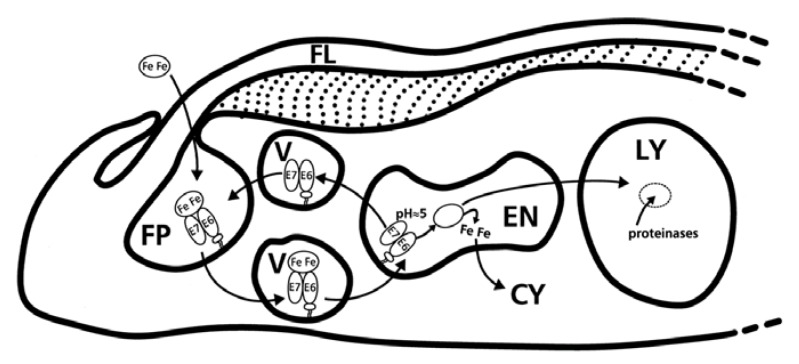
The trypanosomal transferrin receptor is located in the flagellar pocket and is internalized via receptor mediated endocytosis. The transferrin receptor of T. brucei (TbTfR) binds one molecule of Tf [86]. The parasite internalizes host transferrin by transferrin receptor-mediated endocytosis, facilitated by TbRab5. Holo- and apo-transferrin are bound at pH 7 and released at pH 5. Iron is incorporated into the cell cytosol and Tf is degraded by the lysozyme [19]. **Key: E7/E6**, heterodimeric transferrin receptor; **ellipse with Fe**, holo-transferrin; **empty ellipse**, apo-transferrin; **FP**, flagellar pocket; **FL**, flagellum; **V**, endo- and exocytotic vesicles; **EN**, endosome; **LY**, lysosome; **CY**, cytosol. Reprinted from Steverding, D. The transferrin receptor of Trypanosoma brucei. Parasitol. Int. 2000, 48, 191–198 doi:10.1016/S1383-5769(99)00018-5, with permission from Elsevier.

The trypanosomal FP, a membrane invagination surrounding the base of the flagellum, is a specialized organelle with multiple roles in the trypanosome [91,92]. This region is uniquely excluded from the sub-pellicular microtubule array under the parasites’ plasma membrane [93,94]. The FP is also delineated from the rest of the plasma membrane by the FP collar, an electron-dense annulus, without which the FP is lost [93,95]. This collar encloses the FP lumen, a space filled with a carbohydrate-rich matrix with a poorly defined composition and unknown function [91]. The FP is the main turnover point for parasite nutrition [91]. As the only site of exo- and endocytosis by the trypanosome, the FP is part of a multi-organelle intracellular complex comprised of the Golgi complex, the endoplasmic reticulum as well as secretory and endocytic organelles, making it an important cog in the trypanosome’s virulence and protein trafficking [92,95]. The efficiency of the FP protein trafficking is comparable to that of mammalian cells, which is quite remarkable, given that it covers about 2–5% of the total surface of the trypanosome [88]. In BSF trypanosomes, the FP is a site of high protein trafficking, with infectivity tied closely to a high rate of endocytosis [95]. Efficient nutrient scavenging occurs in the FP via selective retention of many of the invariant or variant host-associated nutrient receptors within its lumen by yet-unknown mechanisms but, mostly postulated to be the dynamic result of the high endocytic rate [95]. It is within the FP that the trypanosomal TfR binds iron laden transferrin (holo-Tf) as well as iron free transferrin (apo-Tf) as a GPI-anchored heterodimeric complex (Figure 6) [57,86].

The TfR–ligand complex is endocytosed via a clathrin-dependent pathway [12,96]. Invagination of clathrin-coated vesicles leads to internalization of the receptor–ligand complex and subsequent discharge into the intracellular tubular system [95,96]. The endocytosis process has been hypothesized to involve the cleavage of the intracellular GPIs by the GPI-phospholipase leading to production of DAG and inositol-phosphoglycan [97,98,99]. DAG is an intracellular second messenger for signaling in eukaryotes [97]. Its role in stimulating endocytosis of Tf in the BSF trypanosome is proposed to be an adaptation of *T. brucei* to compete effectively with the mammalian host cells for Tf, as it does not have the same effect in mammalian Tf endocytosis [97]. Binding of DAG to its cognate receptors leads to their activation with the subsequent downstream activation of the protein tyrosine kinase (PTK)-dependent DAG signaling pathway [97]. The PTK is responsible for the phosphorylation and activation of the other proteins of the endocytic system including clathrin, actin, adaptins, and other components [97]. 

Once in the endosome, the acidic pH (6.5–4.5) enhances the release of the iron bound to the holo-Tf:TfR complex leading to formation of apo-Tf:TfR complex (Table 2) [57,86]. However, at the low (acidic) pH, the trypanosomal TfR, in contrast to the mammalian TfR, loses affinity for apo-Tf [19]. The apo-Tf is in turn delivered to the lysosomes for proteolytic degradation by the *T. brucei* cathepsin B-like protease (TbcatB) [100,101]. The resulting fragments are transported out of the cell via the TbRAb11 positive recycling vesicles [102]. TbRAB11 is specifically present in endosomal structures with recycling cargo molecules [102].

The iron released from holo-Tf is initially converted from Fe^3+^ (insoluble) to Fe^2+^ (soluble) via two ferric reductases, i.e., a cytochrome b561-type (Tb927.6.3320) and a NADPH-dependent flavoprotein (Tb11.02.1990), before being imported into the cytoplasm in cooperation with the divalent cation transporter, *T. brucei* Mucolipin-like protein (TbMLP) [105]. The TbMLP, a protein of the endocytic system, is expressed both in the bloodstream and insect stages of the parasite, with high expression in the lysosomes [105]. However, other iron transport mechanisms such as ferric reductase and putative divalent metal transporters containing ZIP domains might also be involved [105,106]. Once in the cytosol, it is presumed that excess iron is stored in a storage compartment and released when cytosolic iron levels decline, which in turn is a possible signal for the TfR upregulation [107]. 

During periods of acute iron scarcity (which has only been documented using in vitro cultivated trypanosomes), mirroring those expected during the switch from one host to another or during chronic infection in the mammalian host, it has been shown that the TfR can be found outside of the FP [107]. The receptor then exists as islands within the VSG coat and was initially hypothesized to allow the parasite to utilize a wider surface area to capture any Tf [61,71]. This spillover is precipitated by an approximately 3- to 5-fold upregulation of the transcription of the receptor [61]. The upregulation of the *TfR* gene is not triggered by an increase in serum concentrations of the apo-Tf, as the receptor does not discriminate between holo- and apo-Tf [68,108]. Rather, upregulation has recently been shown to be mediated via the 3’ untranslated region (UTR) of the *TfR* gene that gets activated upon a reduction in the cytosolic iron concentration [107,109]. This signal triggers the upregulation long before the depletion of the iron stores in the parasite, thus allowing division of the parasite with the subsequent cycling and selection of a suitable high affinity TfR from the ESAGs [107]. The TfR has also been modelled to spread out from the surrounding VSG molecules, which previously was presumed to allow adequate contact and capture of Tf [74]. However, this hypothesis has recently been contradicted by evidence that the TfR outside of the FP is not functional, as it is composed of an ESAG6 homodimer, rather than an ESAG6/7 heterodimer [110]. The GPI valence in trypanosomes has been shown to be a critical determinant of intracellular sorting, with molecular complexes with two GPIs (GPI^2^) being trafficked out of the FP, one GPI (GPI^2^) being retained within the FP, and non-GPI-anchored complexes being degraded in the lysosome [110]. Given that ESAG6 is the GPI-anchored partner in the complex, when over-expressed as homodimer during iron starvation, its GPI valence allows its escape from the FP [110].

## 5. The Trypanosomal Transferrin Receptor as A Target for Chemotherapeutic Purposes

Given the differences between the mammalian and the trypanosomal iron uptake in the form of Tf, it seems feasible to selectively target this pathway for chemotherapeutic purposes [106]. The mammalian host-trypanosome interaction is characterized by a macrophage hyper-activation, which through enhanced erythrophagocytosis cascades to anemia [111,112]. This iron deficiency represents a key challenge to the BSF trypanosomes, a situation further exacerbated by the release of various cytokines and hormones, such as the hepatocyte-derived hepcidin [113]. Hepcidin helps to further accentuate iron deficiency, by down-regulating the iron-exporting ferroportin-1, limiting the contribution of cellular iron to the blood [56,113]. As the parasite has to survive in already limiting conditions, interfering with its otherwise efficient scavenging of iron from the host, may represent a new strategy for treatment of AT [84].

The use of iron chelators to deprive parasites of iron and therefore limit the parasites’ growth within the mammalian host represents an interesting chokepoint [56,63,106,114]. In another haemo-protozoan parasite, *Plasmodium*, the iron metabolism has been a successful in vivo target for many compounds, with iron chelation being a consistently applied therapy [115,116,117]. One iron chelator in particular, a desferrithiocin analogue, has been already applied in human trials for mitigation of iron mediated damage in transfusional iron overload [118,119]. While not curative in nature, the chelation of iron from the incorporation into apolipoproteins or even into Fe^3+^-containing enzymes, e.g., ribonucleotide reductase, by chelators such as deferoxamine, has been shown to limit in vitro the growth of trypanosomes [104,120]. Given that the BSF parasite population needs to rapidly divide to keep up with the high rate of clearance by the immune system, this approach seems useful [121]. However, the use of these chelators is limited by both their water solubility, as well as their cytotoxicity, when applied in vitro on mammalian cells [120]. The potential for these drugs, however, is in the reduction of these unsuitable traits, e.g., by making lipophilic iron-chelating agents with reduced toxicity and in using these in conjunction with currently available anti-trypanosomal drugs [84,120]. 

Blocking the uptake of Tf by selectively targeting the trypanosomal TfR has been proposed severally as an alternative to controlling parasitemia in mammals [58,84]. It has already been shown that the TfR can be targeted by antibodies, with the main problem being delivery of the antibodies to the FP in sufficient quantities to achieve a therapeutic effect [57]. The question then arises whether a smaller targeting molecule, such as a single-domain antibody (sdAb) could work to target the receptor and block it. Such sdAbs do exist as nanobodies (Nbs), i.e., nanometer-sized camelid derived single-domain antibody fragments, which have been used for such cryptic targets as beta-lactamase enzyme active sites amongst other targets [122,123,124]. In addition, the specificity of nanobodies for the trypanosomes’ unique and cryptic sites on their surface proteins has been previously applied to deliver toxic molecules into trypanosomes [125]. Hereby, a nanobody against the conserved region of VSG and conjugated to a trypanolytic molecule, i.e., human ApoL1, allowed to target and kill *ˆT. b. brucei* and *T. b. rhodesiense* parasites [125]. In addition, nanobodies against the variable part of VSG have also been successfully raised that block the endocytic machinery or target drugs to the trypanosome thereby causing its lysis and death [126,127]. Despite these properties, no experiments with nanobodies have been published with regards to the Tf uptake. Though Tf uptake is a potentially lucrative target, the BSF parasite in vivo would be surrounded by adequate ligand, even in the anemic state, thus requiring a considerable dose of a very high affinity nanobody to block, appreciably, the trypanosomal TfR. In addition, the rapid in vivo half-life of nanobodies, due to their size (15 kDa), which is below the renal cut-off (>50 kDa), might further hamper their applicability [122]. Yet, this might be circumvented by increasing their in vivo retention time by generating half-life extended constructs [128,129,130].

## 6. Conclusions

Understanding the trypanosomal transferrin receptor promises to provide unique insights into the trypanosome physiology. As an adaptation to the iron-scavenging lifestyle of the parasite, this molecular complex represents an interesting interface between the host and parasite [106]. Though there has been great progress made in unravelling the working mechanisms of this molecule, there has not been a diagnostic application based on the receptor, nor has there been development of chemotherapeutic agents targeting this essential parasite molecule. There are also gaps in understanding the function of this receptor particularly in the in vivo disease state. This is mainly attributed to the lack of suitable models to fit this context. It is of interest to unravel, in particular, the relevance of the TfR for the BSF trypanosome in this context (in vivo disease state), especially when faced by the fact that the *T. vivax* parasite survives just as well in the host bloodstream without any homologue of the *T. brucei* or *T. congolense* TfR. 

Nonetheless, the TfR is a necessary element for the successful *T. brucei* parasitization of the mammalian host. It is also a relatively invariant molecule in comparison to its homologue, the VSG. Specifically targeting this heterodimeric molecule, at least for chemotherapeutic purposes, provides a novel way to deliver trypanocides or even to slow down parasite growth. This would allow parasite clearance by the infected mammalian host’s immune system, thus controlling parasitemia as well as the inflammation that ensues.

## Figures and Tables

**Table 1:** Features of the transferrin receptors (TfR) of *T. brucei* and human cells. [86].

Features	Trypanosome TfR	Human TfR
Subunit organization	Heterodimer of ESAG6 (50–60 kDa) and ESAG7 (40–42 kDa)	Homodimer of 90-kDa subunits
Post-translational modifications	ESAG6: 2–5 N-linked glycans ESAG7: 2–3 N-linked glycans	Per subunit: 3 N-linked glycan 1 Phosphorylation (Ser 24) 1 Acylation (Cys 62)
Membrane anchorage	GPI-anchor at the C-terminus of ESAG6	1 Transmembrane domain per subunit
Copy number per cell	3000	20000–700000
Ligand/receptor stoichiometry	1 Transferrin molecule per heterodimer	1 Transferrin molecule per monomer

Reprinted from Steverding, D. The transferrin receptor of Trypanosoma brucei. Parasitol. Int. 2000, 48, 191–198 doi:10.1016/S1383-5769(99)00018-5, with permission from Elsevier.

**Table 2:** K_d_-values of ligand–receptor complexes for apo- and holo-transferrin at pH 7 and pH 5 [86].

Transferrin Receptor (nM)	Transferrin	pH	K_d_-Value	Reference
T. brucei strain 427	Holo bovine	7	2.6–3.6	[57,69]
		5	12	[19]
	Apo bovine	7	20	[103]
		5	1100	[19]
Human cells	Holo human	7	1.9–7.7	[104]
		5	13	[104]
	Apo human	7	>700	[104]
		5	13–21	[104]

Reprinted from Steverding, D. The transferrin receptor of Trypanosoma brucei. Parasitol. Int. 2000, 48, 191–198 doi:10.1016/S1383-5769(99)00018-5, with permission from Elsevier.
